# Fine-scale geographic variations of rates of renal replacement therapy in northeastern France: Association with the socioeconomic context and accessibility to care

**DOI:** 10.1371/journal.pone.0236698

**Published:** 2020-07-28

**Authors:** Maxime Desmarets, Carole Ayav, Kadiatou Diallo, Florian Bayer, Frédéric Imbert, Erik André Sauleau, Elisabeth Monnet, on behalf of the VIGIE Study Group

**Affiliations:** 1 CIC-1431 INSERM, CHU Besançon, Université de Franche-Comté, Besançon, France; 2 UMR1098 RIGHT, Université Bourgogne Franche-Comté, EFS, INSERM, Besançon, France; 3 CIC-1433 Epidémiologie Clinique, INSERM, CHRU Nancy, Université de Lorraine, Nancy, France; 4 Agence de la Biomédecine, Saint Denis La Plaine, France; 5 Observatoire Régional de la Santé d'Alsace, Strasbourg, France; 6 Laboratoire de Biostatistique, ICube UMR CNRS 7357, Université de Strasbourg, Strasbourg, France; Universita degli Studi di Perugia, ITALY

## Abstract

**Background:**

The strong geographic variations in the incidence rates of renal replacement therapy (RRT) for end-stage renal disease are not solely related to variations in the population's needs, such as the prevalence of diabetes or the deprivation level. Inequitable geographic access to health services has been involved in different countries but never in France, a country with a generous supply of health services and where the effect of the variability of medical practices was highlighted in an analysis conducted at the geographic scale of districts. Our ecological study, performed at the finer scale of townships in a French area of 8,370,616 inhabitants, investigated the association between RRT incidence rates, socioeconomic environment and geographic accessibility to healthcare while adjusting for morbidity level and medical practice patterns.

**Methods:**

Using data from the Renal Epidemiology and Information Network registry, we estimated age-adjusted RRT incidence rates during 2010–2014 for the 282 townships of the area. A hierarchical Bayesian Poisson model was used to examine the association between incidence rates and 18 contextual variables describing population health status, socioeconomic level and health services characteristics. Relative risks (RRs) and 95% credible intervals (95% CrIs) for each variable were estimated for a 1-SD increase in incidence rate.

**Results:**

During 2010–2014, 6,835 new patients ≥18 years old (4231 men, 2604 women) living in the study area started RRT; the RRT incidence rates by townships ranged from 21 to 499 per million inhabitants. In multivariate analysis, rates were related to the prevalence of diabetes [RR (95% CrI): 1.05 (1.04–1.11)], the median estimated glomerular filtration rate at dialysis initiation [1.14 (1.08–1.20)], and the proportion of incident patients ≥ 85 years old [1.08 (1.03–1.14)]. After adjusting for these factors, rates in townships increased with increasing French deprivation index [1.05 (1.01–1.08)] and decreased with increasing mean travel time to reach the closest nephrologist [0.92 (0.89–0.95]).

**Conclusion:**

These data confirm the influence of deprivation level, the prevalence of diabetes and medical practices on RRT incidence rates across a large French area. For the first time, an association was found with the distance to nephrology services. These data suggest possible inequitable geographic access to RRT within the French health system.

## Introduction

Variations in incidence rates of renal replacement therapy (RRT) for end-stage renal disease (ESRD) between and within countries are well documented [1,2], but the respective roles of the underlying factors involved are difficult to disentangle [3,4]. In a given population, the RRT incidence is the result of several demand-side factors (largely dependent on demographics and morbidity) and a set of supply-side factors, which are themselves shaped by the available resources in the health system [5].

In France, despite universal health coverage and a generous supply of health services, the national Renal Epidemiology and Information Network (REIN) registry has highlighted noticeable differences between and within regions in the incidence rates of RRT [6–8]. A first ecological study, performed at the relatively broad geographical scale of the district (French *départements*), showed that in addition to the prevalence of diabetes, several contextual socioeconomic factors and medical practice patterns were involved in the incidence disparities between 85 metropolitan districts during 2008–2009 [4]. These results raise the question of possible social and territorial discrepancies in dialysis access at a time when French health authorities are launching a national health strategy to tackle healthcare inequalities [9].

To better control possible ecological bias, we set up a study at a finer resolution scale (township; French *canton*) in a northeastern French area of more than 8 million inhabitants, where neighboring areas have shown noticeable disparities in the incidence of RRT [6]. We aimed to 1) map the incidence rates of RRT at the township scale and 2) investigate, at this geographic scale, the association between age- and sex-adjusted incidence rates and the socioeconomic environment as well as geographic accessibility to healthcare after adjusting for morbidity and mortality levels and medical practice patterns. Our approach was based on a conceptual model inspired by the Caskey et al. framework [5], in which the incidence of RRT is related to the burden of chronic kidney disease in the population, socioeconomic environment, accessibility to primary and secondary care and medical practices in dialysis.

## Materials and methods

### Design and setting

This ecological study was conducted at the township scale in two administrative French regions (Grand Est and Bourgogne Franche-Comté) comprising 18 districts and 282 townships and totaling 8,370,616 inhabitants as of December 31, 2014.

### Patients and individual-level variables

The patients were all new patients ≥ 18 years old living in the two regions and registered in the national REIN registry [10] as having started RRT (dialysis or renal transplantation as first treatment) between January 2010 and December 2014. Patients were grouped by township according to their place of residence at the time of RRT initiation, and baseline characteristics (age, sex, initial treatment conditions) were compared by township.

### Townships and contextual variables

**Fig 1:**
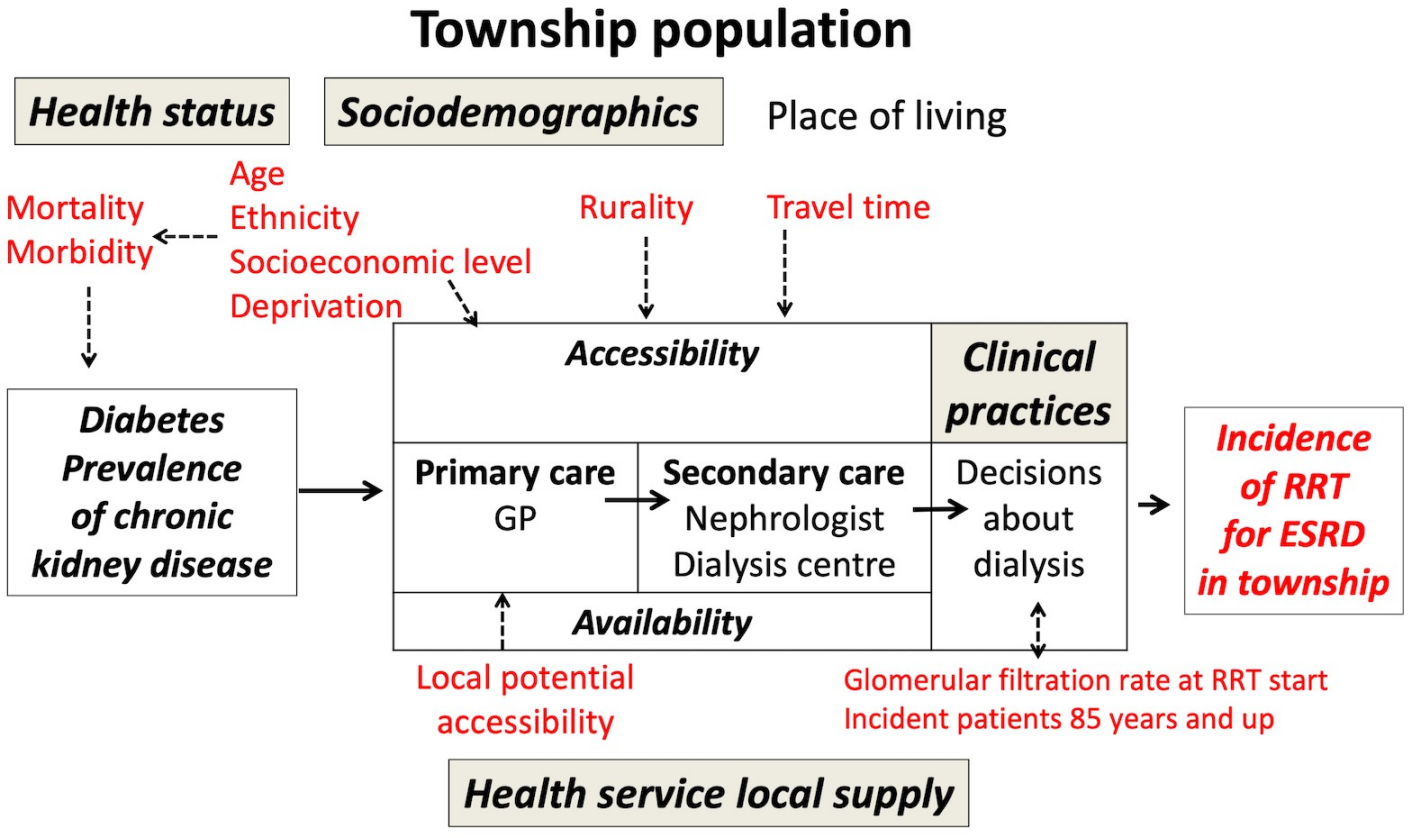
Underlying theoretical epidemiological model adapted from Caskey et al [5]. Indicators considered in the analysis are in red. GP = general practitioner; RRT = renal replacement therapy; ESRD = end-stage renal disease.

The median population in the 282 townships was 21,478 (range 6,556 to 274,394; 2012 estimates). With reference to previous studies [5,11–15], we built a conceptual model considering a set of 18 indicators covering the three following domains (Fig 1): the population health status, the sociodemographic context and the health service characteristics.

The full list of these indicators with the data sources is available in the supporting information (S1 Table). All indicators were available at the township level, except indicators of medical practices in dialysis, which were available only at the district level.

Population health indicators were comparative figures of premature, cardiovascular, and diabetes-related mortality as well as comparative prevalence figures of treated chronic illnesses and treated diabetes. Indeed, the burden of chronic kidney disease could be related to general population health level and to cardiovascular mortality, whereas diabetes prevalence could explain a substantial part of the variability in ESRD incidence [4].

Demographic indicators were the proportion of foreign-born people (a surrogate marker of ethnic origin) and the proportion of people living in rural areas, where service remoteness could decrease healthcare accessibility. To calculate percentages of people living in rural areas, we used the National Institute of Statistics and Economic Studies (INSEE) 2010 classification, distributing the municipalities into either predominantly urban or predominantly rural municipalities [16]. Since living in a more or less privileged context may influence both ESRD incidence and healthcare accessibility, the socioeconomic indicators considered were educational level, distribution of occupational classes in the working population and unemployment rate. These indicators, available from national census data, were considered separately and were then combined according to the FDep deprivation index. This index, validated in France at the municipality scale in 2009 [17], combines the three census-based indicators with income data provided by tax authorities. Indeed, the FDep index is the first component of a principal component analysis of four variables: the median household income, the proportion of high school graduates in the population ≥ 15 years old, the proportion of blue-collar workers in the working population and the unemployment rate. Whereas the first two variables constitute negative dimensions of deprivation, the last two constitute positive dimensions, and the index value increases as deprivation increases [17].

Health service indicators covered accessibility to primary and secondary care as well as clinical practices in dialysis. As chronic kidney disease (CKD) screening is a part of primary care and general practitioners (GPs) play a gatekeeping role for CKD patients, we considered the local potential accessibility (LPA) indicator [18], which measures real primary-care availability, taking into account both supply and demand in township municipalities. For geographic accessibility to secondary care, we used indicators of travel time to the closest nephrologist and dialysis unit. Travel times by car to reach the closest nephrologist or the closest dialysis unit (in minutes) were computed for each municipality of the study area by using a distance matrix based on the national French road dataset [BD TOPO 2015, French national geographical and forest information institute (IGN)], weighted by road classification, topology and demographic context such as population density and land use (INSEE and CORINE Land Cover inventory). These calculations used the geolocated data of municipality town halls and nephrologist services or dialysis centers. Therefore, distance calculations assumed that inhabitants of a municipality were located at the municipality’s town hall. We computed mean travel times for the inhabitants of each township, corresponding to the average travel time for all municipalities belonging to the township and weighted by the number of inhabitants of each municipality. Finally, we considered three indicators illustrating possible differences in clinical practices in dialysis used in previous French studies: the median estimated glomerular filtration rate (eGFR) at RRT start, the percentage of incident patients 85 years and older and the percentage of incident patients who died within 3 months [4,19].

### Ethics statement

This retrospective study was approved by the French Biomedicine Agency in 2015 and included patients’ data that were deidentified directly in the REIN database before extraction for the analysis. The French REIN registry was approved by the French data protection agency [*Commission Nationale de l’Informatique et des Libertés* (CNIL)] in 2010. REIN is registered with the CNIL under the following number: 903188 Version 3.

### Statistical analysis

#### Incidence rate in townships and associations with patients’ characteristics and contextual variables

RRT incidence rates were estimated for the whole area and for the 282 townships by using the number of inhabitants who started RRT from 2010 to 2014, with the corresponding person-year denominators calculated from the population estimates for 2012 (provided by INSEE). We calculated age- and sex-standardized (2005 EU-27 population [20]) incidence rates per million inhabitants, categorized them into quintiles and then mapped them at the township scale. Patient characteristics by township and contextual variables were averaged within each incidence-rate quintile, and obtained values were compared using a trend test. P<0.05 was considered statistically significant. Maps at the township scale were produced for significant contextual variables to depict their geographical distributions.

#### Spatial analysis and hierarchical Bayesian Poisson modeling

Considering the fine resolution scale (township) and the variability of RRT incidence rates among townships, we performed a spatial analysis by using standardized incidence ratios (SIRs), the ratio of the number of cases observed in a township (O) to the number of expected cases (E). The latter is calculated by applying the age- and sex-specific incidence rates of the whole area to the corresponding number of person-years at risk during the study period in the township. The heterogeneity of SIRs was assessed with the coefficient of variation and the Potthoff-Whittinghill test. Autocorrelation in neighboring townships was tested with Moran’s I statistic using binary adjacency for describing the relationship between townships (weight is equal to 1 if townships are adjacent and is equal to zero otherwise) [21]. Thus, we used a hierarchical Bayesian Poisson model [22] to produce smoothed relative risk (RR) estimates (with 95% Bayesian credibility interval [CrI]) by township. In this model, the number of observed cases in township i (O_i_) follows a Poisson distribution whose mean is R_i_E_i_, where R_i_ is the RR in township i and E_i_ is the number of expected cases. The regression model assumes that the natural logarithm log(R_i_) is a sum of effects, one describing unstructured heterogeneity of the SIRs and one corresponding to the local spatial autocorrelation [22]. Univariate analysis was then performed, integrating each contextual variable into the regression model. The RR was obtained from the parameter exponent (point estimate = median value of the posterior probability distribution) and is expressed as the RR of the incidence of RRT associated with an increase of 1 SD in the level of each variable. Covariables whose 95% CrI for the RR did not include 1 were considered significantly associated with the risk in townships. Finally, stepwise regression was performed by forward selection, adding successively the variables giving the best improvement of the deviance information criterion (DIC) [22]. In the final model, the DIC change after the deletion of spatial components was measured to assess the fraction of spatial variability embedded into retained contextual variables.

Analyses were performed with SAS v9.4 and the R packages DCluster [23] and R2WinBUGS [24]. The Bayesian model inference was estimated in WinBUGS [25] with Markov chain Monte Carlo (MCMC) methods. Vague distributions were chosen for prior distributions, and sensitivity analyses were conducted to assess the effect on parameter estimations. The convergence of MCMC parameter estimates was assessed as proposed by Brooks and Gelman [26]. Maps by township were produced with QGIS [27]. Source map data (GEOFLA v2.2) were provided by IGN under Open Licence v2.0 (compatible CC-BY). The data were modified to reflect the level of aggregation used for the contextual indicators.

## Results

During 2010–2014, 6,835 new patients ≥18 years old (4231 men, 2604 women) started RRT in the 282 townships in northeastern France; the age- and sex-standardized RRT incidence rate was 183.1 per million inhabitants (pmi) (95% confidence interval [95% CI] [178.7–187.5]). The mean (± SD) age at RRT start was 69 ± 15 years; 152 (2.2%) patients started with a pre-emptive transplant and 6,683 (97.8%) started with dialysis, 5,494 (80.3%) started with hemodialysis and 1,189 (17.4%) started with peritoneal dialysis.

**Fig 2:**
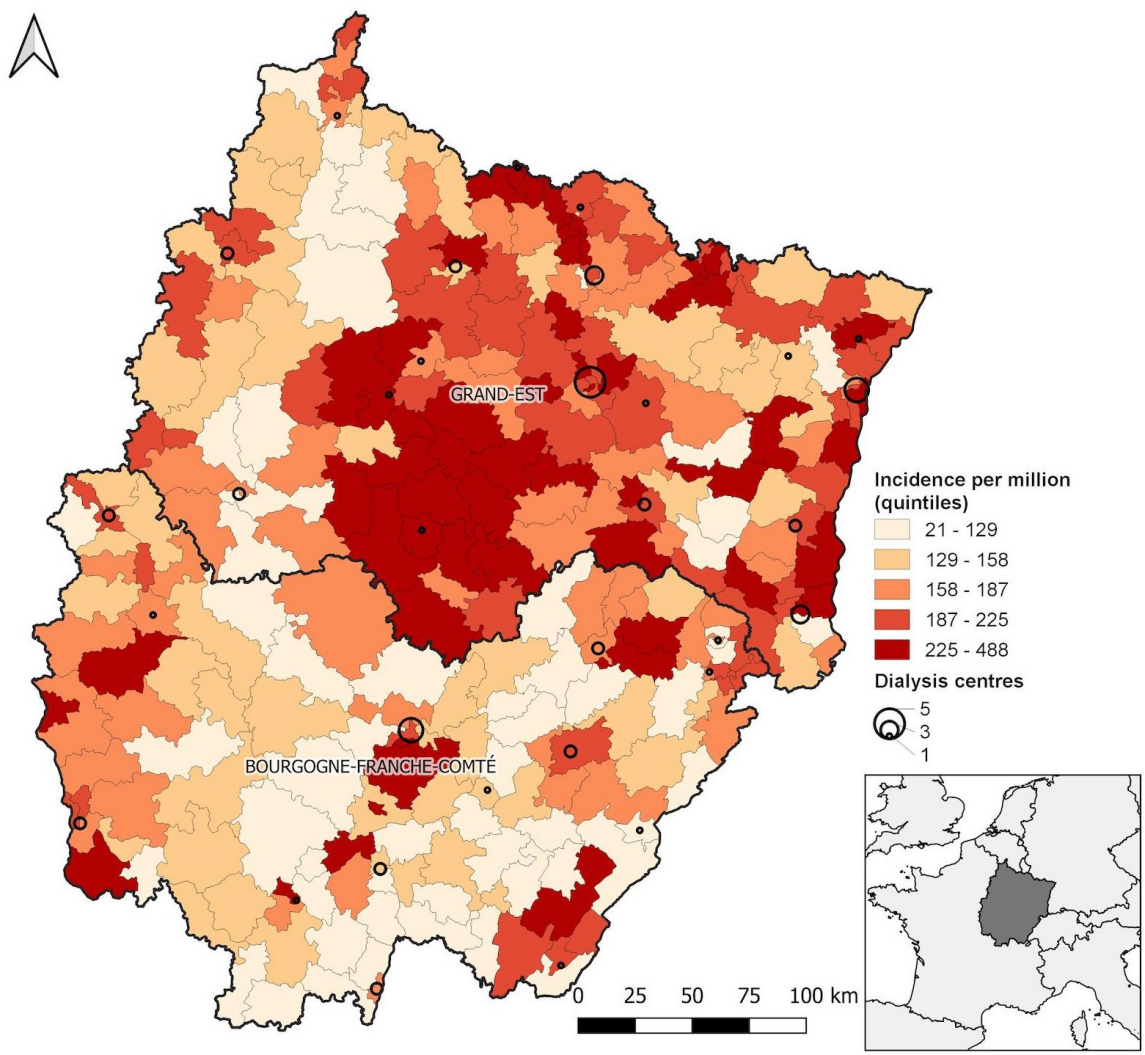
Map of age- and sex-adjusted incidence rates of renal replacement therapy in 282 townships of northeastern France [2010–2014, standard = EU 27 population (2005)]. Categories are quintiles. Circles represent the number of dialysis centers.

Age- and sex-standardized RRT incidence rates varied markedly across townships, from 21.5 [95% CI 0–51.3] to 488.2 [283.6–692.8] pmi (Fig 2).

Patient characteristics and contextual variables for the 282 townships as a whole and by quintiles of RRT incidence are shown in Table 1.

**Table 1:** Mean characteristics of patients who started RRT for end-stage renal disease and mean values of the contextual indicators in townships for the whole area and by quintiles of RRT incidence rate.

		RRT incidence rate^a^ quintiles, pmi					
	Whole area	< 126	126–157	158–186	187–224	> 224	P value^b^
Number of townships	282	56	56	57	56	57	
Patients n (%)	6835 (100)	540 (7.9)	886 (13.0)	1279 (18.7)	2024 (29.6)	2106 (30.8)	
**Mean patient characteristics**							
Age at RRT start (years)	68.5±4.3	67.7±6.0	67.9±3.8	68.1±4.4	69.1±3.5	69.5±3.3	0.008
Male sex (%)	63.2±14.5	64.0±21.3	65.7±14.6	65.1±13.9	60.1±10.6	61.1±8.8	0.06
Treatment at RRT start (%)							
• Hemodialysis	79.6±14.0	76.2±19.5	76.6±12.9	81.3±12.3	79.9±11.4	83.5±11.5	0.003
• Peritoneal dialysis	20.5±13.0	28.0±19.6	21.4±10.6	19.0±10.4	19.0±10.6	15.9±9.9	<10^−4^
**Mean township characteristics**							
**Population health status**							
• Premature^c^ mortality ^d^	103.1±17.7	99.6±16.0	103.0±19.0	107.3±21.2	100.3±15.7	105.0±15.0	0.27
• Cardiovascular mortality ^d^	109.2±14.2	108.0±13.7	111.0±13.4	111.3±13.6	105.4±14.0	110.1±15.8	0.80
• Diabetes-related mortality ^d^	124.1±35.3	121.6±36.8	126.3±33.1	126.8±37.3	120.4±34.6	125.5±35.4	0.90
• Prevalence^e^ of all treated chronic illnesses	95.3±13.3	89.3±9.2	98.3±11.6	93.7±13.5	97.8±15.4	97.4±13.9	0.004
• Prevalence^e^ of treated diabetes	105.5±17.3	94.5±10.8	107.1±12.6	102.2±15.8	111.1±17.6	112.6±21.3	<10^−4^
**Demographic-socioeconomic context**							
• Foreign-born people (%)	2.8±3.5	2.5±2.6	2.6±3.4	2.9±4.3	2.8±3.4	2.9±3.7	0.49
• People living in a rural area (%)	47.3±32.9	57.1±31.1	59.2±30.0	45.8±30.2	31.9±30.8	42.6±35.6	<10^−4^
• Adults without a high school diploma (%)	65.3±6.4	64.9±6.0	66.4±5.1	65.0±6.5	63.4±8.1	66.7±5.7	0.84
• Blue-collar workers^f^ (%)	28.7±5.9	28.2±5.5	29.4±4.7	28.0±6.2	28.0±7.5	29.6±5.1	0.58
• Managers and higher-level professionals^f^ (%)	10.3±4.3	10.2±3.8	9.3±2.7	10.8±4.5	12.1±5.4	9.5±3.8	0.42
• Unemployment rate (%)	12.1±3.3	10.4±2.4	11.2±2.8	12.7±3.5	12.9±3.4	13.3±3.6	<10^−4^
• FDep index^g^	0.0±1.5	-0.33±1.4	-0.03±1.3	0.04±1.6	-0.14±1.6	0.46±1.5	0.02
**Health services accessibility**							
• Local potential accessibility to general practitioner^h^	64.8±15.3	59.1±11.5	62.3±15.3	64.4±14.3	69.8±14.8	68.5±17.9	<10^−4^
• Mean travel time to closest nephrologist (min)	27.4±13.6	29.9±13.7	31.0±13.8	26.8±12.5	23.2±13.7	26.0±13.3	0.006
• Mean travel time to closest dialysis unit (min)	23.4±11.7	26.2±12.8	25.9±11.7	24.0±12.4	19.6±11.1	21.5±9.3	0.001
**Medical practices in dialysis**							
• Median eGFR at RRT start (mL/min/1.73m^2^)	8.8±1.5	8.0±1.2	8.4±1.2	8.7±1.5	9.1±1.3	9.9±1.7	<10^−4^
• Incident patients ≥ 85 years old (%)	9.7±2.6	8.3±2.3	9.5±2.4	9.5±2.6	9.9±2.3	11.4±2.6	<10^−4^
• Incident patients who died within 3 months (%)	8.2±2.5	7.0±2.3	7.9±2.6	8.1±2.6	9.0±2.5	9.0±2.3	<10^−4^

Values expressed with ± sign are the mean ± SD. RRT = renal replacement therapy, pmi per million inhabitants, eGFR = estimated glomerular filtration rate.

^a^ age- and sex-standardized (2005 EU 27 population [16]).

^b^ trend test.

^c^ all deaths before age 65.

^d^ comparative mortality figure, standard = 100 French population 2006.

^e^ comparative prevalence figure, standard = 100 whole French population (2006).

^f^ percentage among active population.

^g^ France Deprivation index, according to Rey et al [17].

^h^ according to Barlet et al [18].

The mean proportion of patients starting with peritoneal dialysis significantly decreased from the first to the fifth RRT incidence quintile (p<10^−4^) and was reversed for patients starting with hemodialysis (p = 0.003). A significant trend across incidence quintiles was observed between prevalence figures for all treated chronic illnesses and for treated diabetes (p = 0.004 and p<10^−4,^ respectively), the proportion of the population living in rural areas (p<10^−4^), the unemployment rate (p<10^−4^), the FDep index (p = 0.02), the LPA to GPs (p<10^−4^), and the travel times to the closest nephrologist and to the closest dialysis unit (p = 0.006 and p = 0.001, respectively), so that townships with a higher prevalence of all treated chronic illnesses or of treated diabetes, a lower proportion of people living in rural areas, a higher unemployment rate, a higher mean FDep index value, and shorter travel times to the closest nephrologist and to the closest dialysis unit had higher RRT incidence rates. Compared with townships with the lowest RRT incidence rates, those with the highest rates featured a significantly higher median estimated glomerular filtration rate (eGFR) at dialysis initiation and higher proportions of incident patients ≥ 85 years old and incident patients who died within 3 months (p <10^−4^, Table 1). The maps depicting the geographical distribution of significant contextual variables are available in the supporting information (S1 Fig)

### Spatial analysis and Bayesian hierarchical modeling

SIR values ranged from 0.13 to 2.50 among townships, giving a coefficient of variation of 37.9%, and the Potthoff-Whittinghill test showed significant heterogeneity (p = 0.004). Moran’s I statistic revealed significant spatial autocorrelation of SIRs (p<10^−4^). Applying the Bayesian model, including both unstructured heterogeneity and spatial autocorrelation, improved the fit of the model, with a DIC decrease of 323.4. Contrasts between townships were reduced, with smoothed RRs ranging from 0.55 to 1.67 (S2A and S2B Fig).

In unadjusted analysis, we found an increased RRT incidence rate in townships with increased comparative prevalence figures of all treated illnesses and treated diabetes, with unemployment rate and FDep index, with local potential accessibility to GPs and with median eGFR at RRT start and the proportion of incident patients ≥ 85 years old (Table 2). Conversely, the RRT incidence rate was significantly decreased with an increased proportion of the population living in rural areas and the mean travel time to reach the closest nephrologist or dialysis unit in townships. Applying a forward selection procedure led to a high reduction in model deviance by including the comparative prevalence figure of treated diabetes, mean travel time to reach a nephrologist and median eGFR at RRT start, whereas contributions of the FDep index and the proportion of incident patients ≥ 85 years old were much weaker (S2 Table). According to the final model, an increase of 17.3% of the comparative prevalence figure of treated diabetes was associated with a 5% increase in RRT incidence, each increase of 1.5 mL/min/1.73 m^2^ in the median estimated GFR at RRT start was associated with a 14% increase in incidence, each increase of 2.6% in the percentage of patients 85 years and older was associated with an 8% increase in incidence, and each 1.5-point increase in the mean FDep index was associated with a 4% increase in incidence. Conversely, each 11.7-min increase in the mean travel time to reach a nephrologist was associated with an 8% decrease in RRT incidence (Table 2).

**Table 2:** Relative risks of RRT incidence associated with contextual indicators derived from hierarchical Bayesian Poisson modeling.

	Unadjusted RR^a^ (95% CrI)	Adjusted RR^b^ (95% CrI)
**Population health status**		
Premature mortality rate	1.01 (0.98–1.06)	
Cardiovascular mortality rate	0.98 (0.94–1.01)	
Diabetes-related mortality rate	0.98 (0.94–1.01)	
Prevalence of all treated chronic illnesses^c^	**1.08 (1.03–1.12)**	
Prevalence of treated diabetes^c^	**1.08 (1.04–1.12)**	**1.05 (1.04–1.11)**
**Demographic-socioeconomic context**		
Percentage of foreign-born population	1.00 (0.97–1.04)	
Percentage of population living in rural areas	**0.94 (0.91–0.98)**	
Percentage of adults without high school diploma	1.00 (0.97–1.04)	
Percentage of manual workers	1.01 (0.98–1.05)	
Percentage of managerial and professional occupations	1.00 (0.97–1.04)	
Unemployment rate^d^	**1.07 (1.04–1.11)**	
FDep index^d^	**1.04 (1.00–1.07)**	**1.05 (1.01–1.08)**
**Health services accessibility**		
Local potential accessibility to general practitioner	**1.05 (1.01–1.08)**	
Mean travel time to closest nephrologist^e^	**0.93 (0.90–0.96)**	**0.92 (0.89–0.95)**
Mean travel time to closest dialysis unit^e^	**0.94 (0.91–0.98)**	
**Medical practices in dialysis**		
Median eGFR at RRT start	**1.19 (1.12–1.25)**	**1.14 (1.07–1.20)**
Percentage of incident patients ≥ 85 years old	**1.14 (1.09–1.20)**	**1.08 (1.03–1.14)**
Percentage of incident patients who died within 3 months	**1.06 (1.00–1.12)**	

RRT = renal replacement therapy, RR = relative risk, CrI = Bayesian credibility interval, eGFR = estimated glomerular filtration rate.

Values in bold indicate significance according to CrI.

^a^ derived from the Bayesian model including spatial components RR for a for a 1-SD increase in the level of each indicator.

^b^ derived from the final Bayesian model (S1 Table) RR for a 1-SD increase in the level of each indicator

^c^ because of the high correlation of both indicators (Spearman’s correlation coefficient = 0.71), only the prevalence of treated diabetes was considered in the multivariable model.

^d^ because of the high correlation of both indicators (Spearman’s correlation coefficient = 0.71), only the FDep index was considered in the multivariable model.

^e^ because of the high correlation of both indicators (Spearman’s correlation coefficient = 0.76), only the mean travel time to the closest nephrologist was considered in the multivariable model.

Mapping the model components indicated that components related to explanatory variables captured most of the relative risk contrasts (S2C and S2D Fig), but the removal of the spatial components from the final model still decreased the model fit (S2 Table).

## Discussion

Our study illustrates the high variability of RRT incidence rates within a large area in France and allows the identification of the main underlying factors. In addition to population needs, expressed by diabetes prevalence and to a lesser extent deprivation level, effects related to the health system itself are highlighted. To our knowledge, this is the first time that an association between distance to closest nephrologist and RRT incidence has been shown in France, a country with generous health services supply, especially in terms of the number of dialysis centers per inhabitant [28,29]. Moreover, as already reported [4], medical decisions about RRT had a significant impact.

If factors related to varying RRT incidence rates worldwide are now better known [5], factors related to the heterogeneity of RRT rates within countries remain to be clarified [3]. Several intranational studies have been performed, especially in the United Kingdom [13,14,30] and in France, at the relatively broad scale of districts [4,19] or using smaller scales, either township, in the Nord-Pas-de-Calais region (4,033,000 inhabitants) [7], or census block, in the Bretagne region (3,094,000 inhabitants) [8]. Choosing smaller territorial units improves statistical power and reduces ecological bias by better displaying geographic variations [31,32]. While RRT incidence rates ranged from 86 to 226 pmi across the 85 districts in the Couchoud et al study [4], the range was greater across the 170 Nord-Pas-de-Calais townships (from 38 to 432 pmi) [7] and across our 282 townships (from 21.5 to 488.2 pmi.). While the study in the Nord-Pas-de-Calais region [7] used the same spatial model as ours, considering both the heterogeneity of population sizes and the spatial correlation of rates in neighboring units, the study conducted in Bretagne [8] searched for clusters of high ESRD incidence, obtaining results that cannot be compared to ours. A strength of our study was the inclusion of a large panel of contextual indicators from three main domains, population health status, demographic-socioeconomic context and health service characteristics, including medical practices in dialysis, whereas spatial modeling in the Nord-Pas-de-Calais region considered only township deprivation index [7].

In our study, RRT incidence rates were not correlated with premature or cause-specific mortality rates in townships but were related to treated diabetes prevalence. Such a positive association, already found in several countries with various health systems [4,11,14], shows that diabetes prevalence may be considered a reliable contextual marker of population RRT need.

The association between the demographic-socioeconomic context and RRT incidence rate is more equivocal. Since no indicator describing population ethnic origin was available in France, we attempted to consider the proportion of foreign-born people as a surrogate marker, but we found no association. By contrast, we found associations between the RRT incidence rate and both indicators of rurality and socioeconomic level. In unadjusted analysis, the RRT rate in townships decreased with an increased proportion of people living in rural areas, as was previously observed in France [4]. The incidence of RRT in the United States was found to be higher in rural than in urban counties because of lower access to pre-ESRD care for rural patients with advanced CKD [11,15], whereas in France, the incidence of RRT was negatively correlated with rurality [4]. In our study, rurality in townships was correlated negatively with accessibility to primary care (Spearman’s Rho = -0.41, p<10^−3^) and positively with mean travel time to reach a dialysis unit (Spearman’s Rho = 0.56, p<10^−3^), showing that accessibility to all health services decreased as the proportion of the population living in rural municipalities increased. In multivariate modeling, the rurality indicator did not improve the model fit (data not shown). This finding might indicate that, in contrast with results observed in the United States [11,15], the consequences of living far from a nephrologist for rural patients living with CKD in our study area could outweigh those of low primary-care availability. Nevertheless, confirming such a hypothesis would require further investigations.

As already observed in France [4,7] and in other countries [30,33], we found a positive correlation between RRT incidence rate and deprived socioeconomic context, estimated by unemployment rate or FDep index. In multivariate modeling, the model fit was slightly improved by including either indicator. The FDep index in France is considered a reliable deprivation ecological index, reflecting a major part of the socioeconomic heterogeneity at various geographic scales [17,34]. Thus, we retained it as a better descriptor of the various components of a deprivation context. Poverty, associated with an increased risk of obesity, diabetes and hypertension, is a known individual risk factor for ESRD [35]. The association at the ecological level between deprived areas and increased RRT incidence rates could be related to greater ESRD risk among inhabitants, although a specific effect of the poverty context cannot be ruled out [36].

Our study illustrates that RRT incidence rates result from population needs as well as supply-side factors. As pointed out by the Organisation for Economic Co-operation and Development [31], a significant portion of geographic variations in health care delivery may be due to unequal access to health services. In the United Kingdom, with a quite low number of dialysis centers per million inhabitants [29], an ecological study found a low probability of starting or receiving RRT with a long travel time to access dialysis [13]. Among the different dimensions of healthcare access [37], we focused on geographic accessibility to a GP as well as to a nephrologist and dialysis center. This issue deserves special attention in France because of large geographic variations in physician-to-population ratios [38]. By contrast, the lack of affordability cannot be considered a barrier because French patients with ESRD can all receive 100% reimbursement of costs for necessary healthcare through the *affection de longue durée* (ALD) program of the national statutory health insurance [28]. In our study, in unadjusted analysis, we found a positive correlation between local accessibility to a GP and RRT incidence in townships. However, the variable was negatively correlated with the mean travel time to reach a nephrologist (Spearman’s Rho = -0.33, p<10^−3^) and was not retained in the final multivariate model. Thus, remote townships are not only far from nephrologists and dialysis services but also frequently have shortages of GPs and pre-ESRD care. This observation reinforces the hypothesis of possible unmet health needs due to healthcare underprovision in these areas.

Finally, our results confirmed that medical practice styles could vary across our study area and had a role in geographic variations of RRT incidence rates, with significant effects of median eGFR at RRT start and the proportion of incident patients ≥ 85 years old. The former may represent a decision marker to start dialysis, and the latter may represent a marker of the propensity to refer or accept frail older patients for dialysis. Geographic variations in both indicators could raise the issue of equity in RRT acceptance among places of care. Nevertheless, the decision to start dialysis or to provide conservative care remains debated, particularly for older patients, depending on their clinical conditions and preferences [39,40]. With no data on case-mix, patient preferences and conservative care frequency, the appropriateness of decisions about starting dialysis cannot be assessed.

Our study has several limitations. Despite the smaller size of geographic units, a possible ecological bias cannot be ruled out. We cannot extrapolate individual risks from observed associations for contextual factors, such as distance to a nephrologist or deprivation. Moreover, because of unavailable data on medical practice indicators at the township scale, we used only district-based indicators, which may reinforce ecological error and imply imperfect adjustment for these factors. Finally, our results may feature residual bias related to unmeasured confounders. However, in ecological regressions, residual spatial autocorrelation may be considered a surrogate marker of spatially structured unmeasured confounders [41]. In our final model, the spatial component contribution to DIC reduction was strongly reduced, and mapping this component showed little residual contrast (supplementary material, S2E Fig).

## Conclusions

This study suggests possible inequitable geographic access to RRT in France. Whatever the population needs and medical practice patterns in dialysis, RRT incidence rates were lower in townships with longer travel times to reach a nephrologist. For patients with CKD, the unequal distribution of the provision of medical care by territory might lead to reduced healthcare access, especially for the frailest or most socially deprived patients. Therefore, maintaining a balanced supply of health professionals throughout territory and improving the coordination between dialysis centers and primary-care teams could be key factors in tackling territorial and social inequalities that may affect CKD patients.

## Supporting information

S1 Table Contextual indicators describing population health status, demographic and socioeconomic context, and health service characteristics for the 282 studied townships in France, with data sources. ^a^ all indicators were established at the township level, except those of medical practices in dialysis, which were available only at the district level. ^b^ standard = French population in 2006. ^c^ all deaths before age 65 years. ^d^ French deprivation index, according to Rey et al [17]. ^e^ according to Barlet et al [18].
(PDF)

S2 Table Stepwise procedure for the selection of the best-adapted Bayesian model. DIC = deviance information criterion, Ri = relative risk in township i, α = intercept, Ui = autocorrelation component in township i, Vi = heterogeneity component in township i.
(PDF)

S1 Fig Maps of contextual variables significantly associated with age- and sex-adjusted incidence rates of renal replacement therapy in 282 townships of northeastern France. Categories are quintiles.
(TIFF)

S2 Fig Mapping of standardized incidence ratios (SIRs), smoothed relative risks and the components of the fitted relative risks derived from the final model (model 7). A SIRs = O/E (observed number of cases/expected number of cases). B Smoothed relative risks (from model 2) log Ri = α + U_i_ + V_i._ C Fitted relative risks (from model 7) log Ri = α + U_i_ + V_i_ + βX_i._ D Components of variation from model 7: explanatory variables exp(βXi). E Spatial component from model 7: exp (U_i_).
(TIFF)

S1 Data (XLSX)
