# Supplementary material for: Fine-scale geographic variations of rates of renal replacement therapy in northeastern France: Association with the socioeconomic context and accessibility to care
Source: PLoS One. 2020 Jul 28;15(7):e0236698. doi: 10.1371/journal.pone.0236698 (PMC7386572; doi:10.1371/journal.pone.0236698)
Supplement: S1 Table — a all indicators were established at the township level, except those of medical practices in dialysis, which were available only at the district level. b standard = French population in 2006. c all deaths before age 65 years. d French deprivation index, according to Rey et al [17]. e according to Barlet et al [18]. (PDF) [file pone.0236698.s001.pdf]

S1 Table. Contextual indicators describing population health status, demographic and socioeconomic context, and health service characteristics for the 282 studied townships in France, with data sources.

	**Variables**<br>Indicators[a]	**Data sources**<br>Year
**Population health status**	**Mortality**[b]<br>- Comparative premature[c] mortality figure<br>- Comparative cardiovascular mortality figure<br>- Comparative diabetes-related mortality figure	**Center for epidemiology of medical causes of death (CepiDC)**<br>- 2012
	**Morbidity**[b]<br>- Comparative prevalence figure for all treated chronic illnesses<br>- Comparative prevalence figure for treated diabetes	**National health insurance system (chronic conditions scheme database)**<br>- 2012
**Demographic-socioeconomic context**	**Place of birth**<br>- Percentage of foreign-born population<br>**Rurality**<br>- Percentage of population living in a rural area<br>**Educational level**<br>- Percentage of population without high school diploma in the active population<br>**Occupational class in the active population**<br>- Percentage of blue-collar workers<br>- Percentage of managers and higher-level professionals<br>**Professional activity**<br>- Unemployment rate<br>**Social deprivation**<br>- FDep index[d]	**National Institute of Statistics and Economic Studies (INSEE)**<br>- 2012
**Health services characteristics**	**Geographic accessibility to primary care**<br>- Local potential accessibility to general practitioner[e]	**Institute for Research and Information in Health Economics (IRDES)**<br>- 2010
	**Geographic accessibility to nephrologist and dialysis care**<br>- Mean travel time to closest nephrologist<br>- Mean travel time to closest dialysis unit<br>**Clinical practices in dialysis (district level measures)**<br>- Median eGFR at RRT start<br>- Percentage of incident patients ≥ 85 years old<br>- Percentage of incident patients who died within 3 months	**Renal Epidemiology and Information Network (REIN) Registry**<br>- 2010-2014

eGFR = estimated glomerular filtration rate, RRT = renal replacement therapy
[a] all indicators were established at the township level, except those of medical practices in dialysis, which were available only at the district level.
[b] standard = French population in 2006.
[c] all deaths before age 65 years.
[d] French deprivation index, according to Rey et al [17].
[e] according to Barlet et al [18].
