# Supplementary material for: Fine-scale geographic variations of rates of renal replacement therapy in northeastern France: Association with the socioeconomic context and accessibility to care
Source: PLoS One. 2020 Jul 28;15(7):e0236698. doi: 10.1371/journal.pone.0236698 (PMC7386572; doi:10.1371/journal.pone.0236698)
Supplement: S2 Table — DIC = deviance information criterion, Ri = relative risk in township i, α = intercept, Ui = autocorrelation component in township i, Vi = heterogeneity component in township i. (PDF) [file pone.0236698.s002.pdf]

S2 Table. Stepwise procedure for the selection of the best-fit Bayesian model

Step	Model	DIC
1	log Ri=α	2079.4
2	log Ri=α + U_i + V_i	1756.0
3	Model 2 + median estimated GFR at RRT start	1746.9
4	Model 3 + prevalence of treated diabetes	1736.0
5	Model 4 + mean travel time to reach closest nephrologist	1723.5
6	Model 5 + FDep index	1722.3
7	Model 6 + percentage of incident patients 85 years and older	1723.1
8	Model 7 – (U_i + V_i)	1753.0

DIC=deviance information criterion
R_i=relative risk in township i, α=intercept, U_i=autocorrelation component in township i, V_i=heterogeneity component in township i
