# Supplementary figures and images for: Fine-scale geographic variations of rates of renal replacement therapy in northeastern France: Association with the socioeconomic context and accessibility to care
Source: PLoS One. 2020 Jul 28;15(7):e0236698. doi: 10.1371/journal.pone.0236698 (PMC7386572; doi:10.1371/journal.pone.0236698)

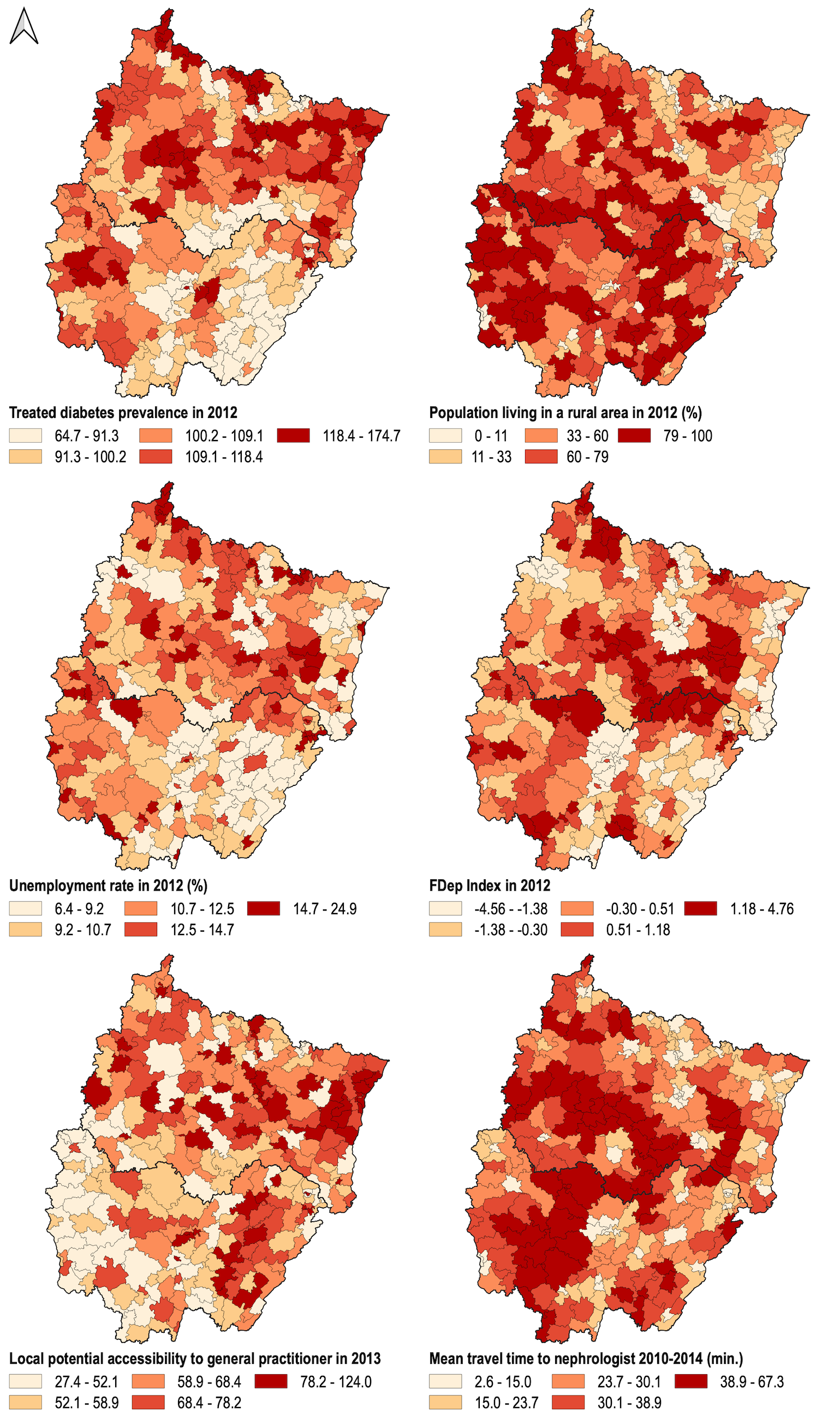

Supplement: S1 Fig — Categories are quintiles. (TIFF) [file pone.0236698.s003.tiff]

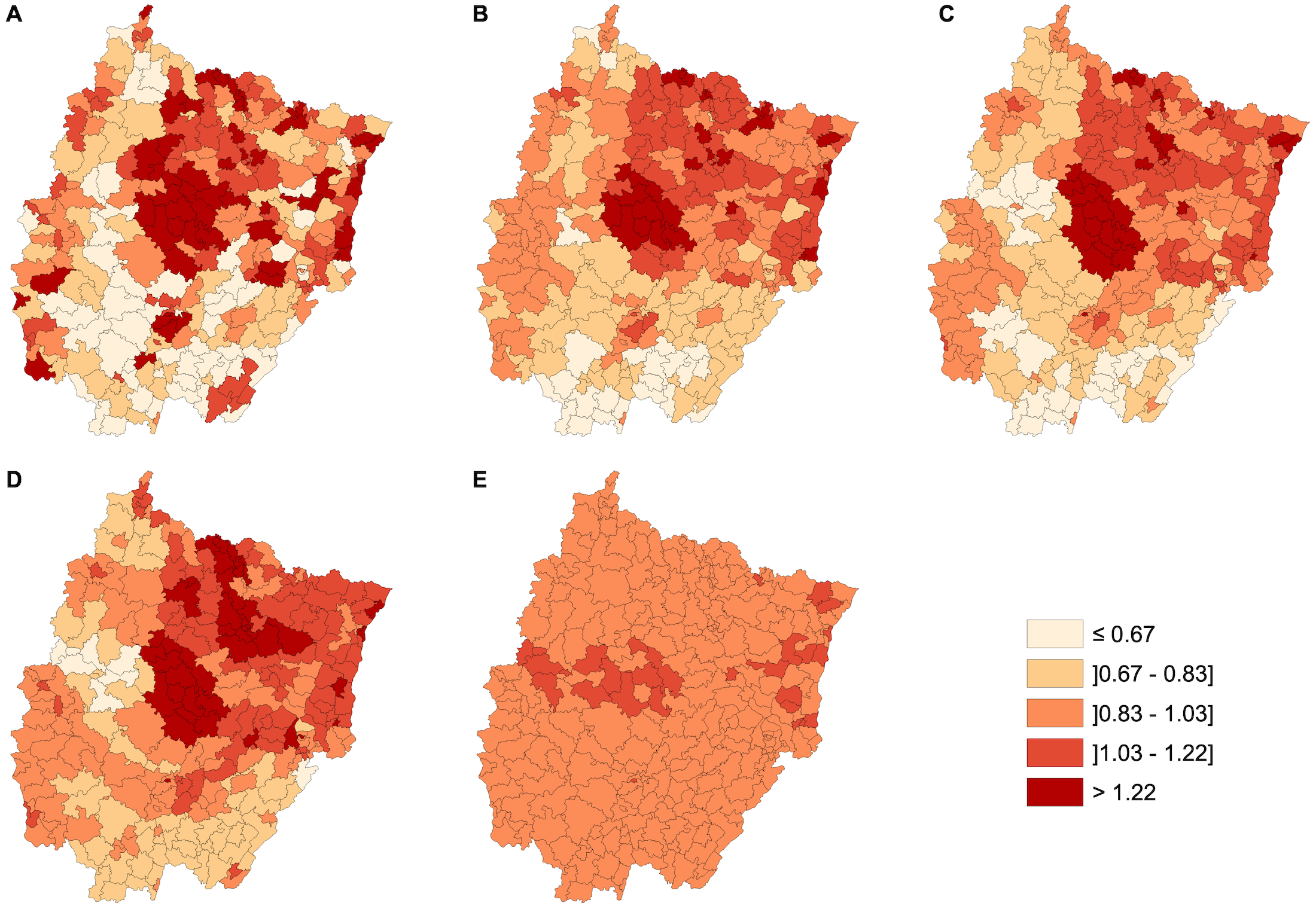

Supplement: S2 Fig — A SIRs = O/E (observed number of cases/expected number of cases). B Smoothed relative risks (from model 2) log Ri = α + Ui + Vi. C Fitted relative risks (from model 7) log Ri = α + Ui + Vi + βXi. D Components of variation from model 7: explanatory variables exp(βXi). E Spatial component from model 7: exp (Ui). (TIFF) [file pone.0236698.s004.tiff]
